# Abscisic Acid-Induced H_2_O_2_ Accumulation Enhances Antioxidant Capacity in Pumpkin-Grafted Cucumber Leaves under Ca(NO_3_)_2_ Stress

**DOI:** 10.3389/fpls.2016.01489

**Published:** 2016-09-30

**Authors:** Sheng Shu, Pan Gao, Lin Li, Yinghui Yuan, Jin Sun, Shirong Guo

**Affiliations:** ^1^Key Laboratory of Southern Vegetable Crop Genetic Improvement, Ministry of Agriculture, College of Horticulture, Nanjing Agricultural UniversityNanjing, China; ^2^Suqian Academy of Protected Horticulture, Nanjing Agricultural UniversityNanjing, China

**Keywords:** abscisic acid, antioxidant enzymes, Ca(NO_3_)_2_ stress, cucumber, grafting, hydrogen peroxide

## Abstract

With the aim to clarifying the role of the ABA/H_2_O_2_ signaling cascade in the regulating the antioxidant capacity of grafted cucumber plants in response to Ca(NO_3_)_2_ stress, we investigated the relationship between ABA-mediated H_2_O_2_ production and the activities of antioxidant enzymes in the leaves of pumpkin-grafted cucumber seedlings. The results showed that both ABA and H_2_O_2_ were detected in pumpkin-grafted cucumber seedlings in response to Ca(NO_3_)_2_ treatment within 0.5 h in the leaves and peaked at 3 and 6 h after Ca(NO_3_)_2_ treatment, respectively, compared to the levels under control conditions. The activities of superoxide dismutase (SOD), ascorbate peroxidase (APX), and peroxidase (POD) in pumpkin-grafted cucumber leaves gradually increased over time and peaked at 12 h of Ca(NO_3_)_2_ stress. Furthermore, in the leaves of pumpkin-grafted cucumber seedlings, the H_2_O_2_ generation, the antioxidant enzyme activities and the expression of *SOD, POD* and *cAPX* were strongly blocked by an inhibitor of ABA under Ca(NO_3_)_2_ stress, but this effect was eliminated by the addition of exogenous ABA. Moreover, the activities and gene expressions of these antioxidant enzymes in pumpkin-grafted leaves were almost inhibited under Ca(NO_3_)_2_ stress by pretreatment with ROS scavengers. These results suggest that the pumpkin grafting-induced ABA accumulation mediated H_2_O_2_ generation, resulting in the induction of antioxidant defense systems in leaves exposed to Ca(NO_3_)_2_ stress in the ABA/H_2_O_2_ signaling pathway.

## Introduction

Greenhouse cultivation is the most common method of vegetable production worldwide. However, secondary soil salinization in greenhouse soil, which is primarily caused by over-irrigation, intensive farming, lack of rain, and excessive application of nitrogenous fertilizers, restricts the development and productivity of vegetables in China (Blanco and Folegatti, 2002; Liang et al., 2005; Yu et al., 2005; He et al., 2007; Daliakopoulos et al., 2016). Several reports have indicated that the characteristics of greenhouse soil are different from those of coastal and inland saline soil. Its main cation is Ca^2+^, and its main anion is ${\mathrm{NO}}_{3}^{–}$, which account for 60 and 67–76% of the total cations and anions, respectively (Yuan et al., 2012; Xing et al., 2015). Excessive Ca(NO_3_)_2_ not only leads to osmotic stress, inhibits biological nitrogen fixation, and modifies microbial soil biodiversity but also causes the formation of reactive oxygen species (ROS) in plants. ROS can disorder the normal physiological metabolism and then inhibit plant growth and decrease crop yield (Sainju et al., 2001). However, plants have evolved adaptive protection mechanisms, such as antioxidant enzymes to protect themselves against the deleterious effects of ROS.

Cucumber (*Cucumis sativus* L.) is one of the most economically important vegetables worldwide, grown both in open fields and protected facilities. According to FAO statistics, the world cultivation area of cucumber was 2,115,457 hm^2^ and the yield was 71,333,414 ton in 2013. In China, these two values were 1,166,690 hm^2^ and 54,362,750 tons, respectively, making China the country with the largest production of cucumber (FAO, 2013). However, the yield of cucumber in China was only 46.6 tons⋅hm^-2^, which was far lower than the highest yield (Netherlands, 666.7 ton⋅hm^-2^), ranking China number 31 in the world. Because of their biological characteristics, cucumber plants are affected by many adverse environmental factors, of which the secondary salinization of greenhouse soil is a major problem, significantly decreasing the yield and quality of cucumber fruits. A common method of adapting plants to environmental stresses is by grafting commercial cultivars onto selected tolerant rootstocks (Lee and Oda, 2002). In horticultural crop production, grafting has already been used for more than 50 years in many regions of the world (Rivard and Louws, 2008). Vegetable seedlings grown under Ca(NO_3_)_2_ stress may possess higher contents of osmotic adjustment substances and higher activities of antioxidant enzymes if they are grafted with selected vigorous rootstocks and suffer less oxidative damage, contributing to a higher production and better quality of fruits (Zhang et al., 2008; Xing et al., 2015).

Abscisic acid has a wide range of physiological functions in higher plants, including regulating plant responses to various adverse environmental factors (Sah et al., 2016). ABA is considered a root-derived signaling molecule. It moves within plants, and its transport plays an important role in determining the endogenous hormone concentrations at the site of action (Seo and Koshiba, 2011). A number of studies suggest that ABA might be the chemical substance responsible for root-to-shoot signaling, especially under abiotic stress conditions (Sah et al., 2016). An increasing body of evidence indicates that one mode of ABA action is related to oxidative stress in plant cells. It is well known that ABA can increase the generation of H_2_O_2_ (Kwak et al., 2003; Laloi et al., 2004); cause the gene expression of superoxide dismutase (SOD), CAT, and APX, increase the activities of these antioxidant enzymes in plant tissues; and enhance the stress resistance of plants (Guan et al., 2000; Jiang and Zhang, 2003; Park et al., 2004; Saxena et al., 2016).

However, little information exists about whether the increased enzyme activities of antioxidants induced by ABA in an H_2_O_2_-dependent way would occur in pumpkin-grafted cucumber seedlings and lead to better plant performance than that in self-grafted cucumber seedlings under Ca(NO_3_)_2_ stress. In this study, the ABA and H_2_O_2_ contents and antioxidant enzyme activities in the leaves of pumpkin-grafted and self-grafted cucumber seedlings under Ca(NO_3_)_2_ stress were examined. In addition, this study investigated whether the increased activities of antioxidant enzymes and their encoded gene (*SOD, POD*, and *cAPX*) expression in pumpkin-grafted cucumber leaves under Ca(NO_3_)_2_ stress are induced by ABA and whether H_2_O_2_ is involved in this induction. The mechanism by which the pumpkin rootstock enhances antioxidant defense for the Ca(NO_3_)_2_ stress tolerance of cucumber seedlings is also discussed.

## Materials and Methods

### Plant Material and Treatments

Cucumber cultivar (*Cucumis sativus* L. ‘Jinyou No. 3’, obtained from Tianjin Kerun Research Institution) was used as the scion. A salt-tolerant pumpkin ‘Qingzhen 1’ (*Cucurbita maxima* × *Cucurbita moschata*, obtained from Qingdao Agriculture Academy of Science) was selected as the rootstock (Xing et al., 2015). Cucumber and pumpkin seeds were surface sterilized with 1% (v/v) sodium hypochlorite, washed thoroughly with distilled water, and then sown in plastic salvers (41 cm × 41 cm × 5 cm) containing quartz sand and incubated in a greenhouse at Nanjing Agriculture University, China. The average day/night temperatures in the greenhouse were at 25–30°C/15–18°C, and the relative humidity was 60–75%. When the scion’s cotyledons were fully expanded and the rootstock’s second true leaves were in the development stage, the insert grafting procedure was performed as described by Lee and Oda (2002). Self-grafted plants were included as the controls. Uniformly sized pumpkin-grafted seedlings were grown hydroponically in plastic containers filled with half-strength Hoagland’s solution (pH 6.5 ± 0.1, EC 2.0–2.2 dS⋅m^-1^) for the next experiments. The solution was replaced every 3 days and continuously aerated with an air pump at an interval of 20 min to keep the dissolved oxygen level at 8.0 ± 0.2 mg⋅L^-1^.

After the full development of the third true leaves, the seedlings were treated as follows: 80 mM Ca(NO_3_)_2_ was added to the solution for the salt stress treatment. Different exogenous substances such as 10 μM ABA, 1 mM sodium tungstate (T, an ABA synthesis inhibitor), 10 mM 1,2-dihydroxybenzene- 3,5-disulfonic acid (Tiron, a specific $O_{2}^{•–}$ scavenger), 100 μM DPI (a specific NADPH oxidase inhibitor) and 5 mM DMTU (a specific H_2_O_2_ scavenger) were used in various treatments for 8 h before salt stress treatment. All of the treatments were as follows:

(a) S-G, self-grafted cucumber seedlings grown in Hoagland’s solution;
(b) S-GN, self-grafted cucumber seedlings grown in Hoagland’s solution with 80 mM Ca(NO_3_)_2_;
(c) P-G, pumpkin-grafted cucumber seedlings grown in Hoagland’s solution;
(d) P-GN, pumpkin-grafted cucumber seedlings grown in Hoagland’s solution with 80 mM Ca(NO_3_)_2_;
(e) P-GN + T, pumpkin-grafted cucumber seedlings pretreated with 1 mM sodium tungstate grown in Hoagland’s solution with 80 mM Ca(NO_3_)_2_;
(f) P-GN + T + ABA, pumpkin-grafted cucumber seedlings pretreated with 1 mM sodium tungstate and 10 μM ABA grown in Hoagland’s solution with 80 mM Ca(NO_3_)_2_;
(g) P-GN + DPI, pumpkin-grafted cucumber seedlings pretreated with 100 μM DPI grown in Hoagland’s solution with 80 mM Ca(NO_3_)_2_;
(h) P-GN + Tiron, pumpkin-grafted cucumber seedlings pretreated with 10 mM Tiron grown in Hoagland’s solution with 80 mM Ca(NO_3_)_2_;
(i) P-GN + DMTU, pumpkin-grafted cucumber seedlings pretreated with 5 mM DMTU grown in Hoagland’s solution with 80 mM Ca(NO_3_)_2_.

The experiments were arranged in a randomized complete block design with three replicates. Each treatment included three containers with 36 seedlings. The third fully expanded leaves (from the top) were sampled at key time points as indicated below and immediately frozen in liquid nitrogen.

### ABA Assay

Fresh leaves were homogenized in an extraction solution containing 80% methanol, 0.05% citric acid and 0.45 mM butylated hydroxytoluene and were then centrifuged at 8,000 × g for 10 min. The samples were dried, and the radioactivity in the pellet was quantified. ABA was assayed using radioimmunoassay as described by Verslues and Bray (2006).

### Measurement of the H_2_O_2_ Level

The H_2_O_2_ content was measured according to the method described by Brennan and Frenkel (1977). The absorbance values via OD at 415 nm were calibrated to a standard graph generated with known concentrations of H_2_O_2_.

### Measurement of the Antioxidant Enzyme Activity

For extract of antioxidant enzymes, fresh leaves were homogenized with 1.6 mL of 50 mM phosphate buffer (pH 7.8) containing 1 mM EDTA and 2% PVP. The homogenate was centrifuged at 12,000 × *g* for 20 min at 4°C, and the resulting supernatant was used to assay enzyme activity as follows: the SOD activity was assayed by monitoring the inhibition of the photochemical reduction of NBT following the method of Giannopolitis and Ries (1997). One unit of SOD activity was defined as the amount of enzyme that was required to cause a 50% inhibition of the reduction of NBT as monitored at 560 nm.

The POD activity was measured according to the method of Rao et al. (1996) with slight modification. For this, 5 mL of the extracted enzyme was mixed with 3 mL of the reaction mixture containing 50 mM PBS (pH 7.0) and 20 mM guaiacol. After pre-incubation at 25°C for 5 min, 6 mM H_2_O_2_ was added to initiate the reaction. Changes in the absorbance at 470 nm within 2 min were recorded to calculate POD activity. One unit of POD activity was expressed as U.g^-1^ FW.

A modified method from Aebi (1974) was used to assay the CAT activity. For this, 100 mL of the extraction was added to 3 mL 50 mM PBS buffer (pH 7.0). After incubation, the reaction was started by the addition of 6 mM H_2_O_2_. The CAT activity was expressed as U.g^-1^ FW.

The APX activity was performed as described by Pinhero et al. (1997). The assay was carried out in a reaction mixture consisting of 50 mM PBS (pH7.0), 0.5 mM AsA, 3 mM H_2_O_2_ and 100 mL of the extraction. One unit of APX activity was defined as an absorbance change of 0.1 unit min^-1^, and the APX activity was expressed as U.g^-1^ FW.

### RNA Isolation and Quantitative Real-Time (qRT-PCR) Analysis

Total RNA was extracted from leaves as described in the TRI reagent protocol (Takara Bio Inc). Primers were designed according to cucumber databases ^1^. and NCBI. Gene specificprimers used for real-time quantitative PCR are provided in the following primers: *SOD*: forward CCTAAACTCTCGTGAATGA and reverse CAGCAGACAAGTATGGATA; *POD*: forward TTGTAATAATGGCGGCTT and reverse GTGTCATAGAAGGTGGAG; *cAPX*: forward TGCTTTCATCACCATCAA and reverse TGTTATGTTCTTGTCTTCCT. *Actin*: forward CCACCAATCTTGTACACATCC and reverse AGACCACCAAGTACTACTGCAC. qRT-PCR was performed on a StepOnePlus^TM^ Real-Time PCR System (Applied Biosystems) using a SYBR^®^ Premix Ex Taq^TM^ II (Tli RNaseH Plus) kit (Takara). The PCR reactions were carried out in triplicate and the thermocycler conditions, 95°C for 30 s, followed by 40 cycles of 95°C for 5 s, 60°C for 30 s, and a final extension of 30 s at 60°C. Relative expression was calculated according to the 2^-ΔΔ^*^C^*^T^ method, the relative gene expression level was normalized against *actin* (the internal standard gene).

### Statistical Analysis

All data were statistically analyzed with SAS 13.0 software (SAS Institute, Inc., Cary, NC, USA) using Duncan’s multiple range test at the *P <* 0.05 level of significance.

## Results

### Effects of Ca(NO_3_)_2_ Stress on the Contents of ABA and H_2_O_2_ in the Leaves of Pumpkin-Grafted and Self-Grafted Cucumber Seedlings

**FIGURE 1:**
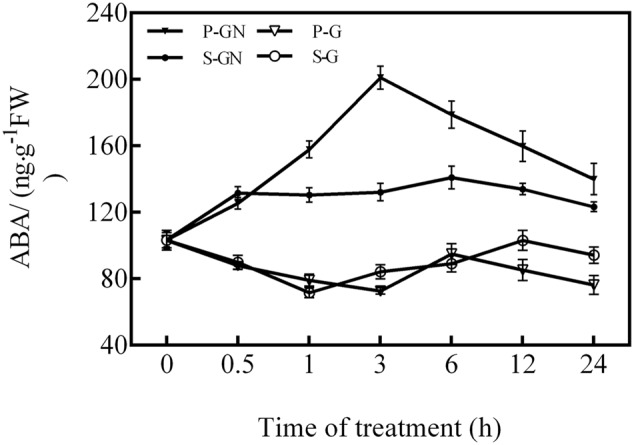
**Time course of changes in the ABA content in the leaves of self-grafted and pumpkin-grafted cucumber seedlings exposed to 80 mM Ca(NO_3_)_2_.** Each value is the mean ± SE of six independent experiments (*n* = 6). P-G, pumpkin-grafted cucumber seedlings grown in Hoagland’s solution; P-GN, pumpkin-grafted cucumber seedlings with 80 mM Ca(NO_3_)_2_; S-G, self-grafted cucumber seedlings grown in Hoagland’s solution; S-GN, self-grafted cucumber seedlings with 80 mM Ca(NO_3_)_2_.

Compared to their corresponding controls, the ABA content in the leaves of pumpkin-grafted and self-grafted seedlings significantly increased under 80 mM Ca(NO_3_)_2_ stress (**Figure 1**), whereas the ABA level in the rootstock-grafted leaves was higher than that of the self-grafted leaves under Ca(NO_3_)_2_ stress. The ABA content in the leaves of pumpkin-grafted cucumber seedlings increased after 0.5 h of Ca(NO_3_)_2_ stress and peaked at 3 h. However, the ABA content in the leaves of the self-grafted cucumber seedlings did not significant changes after the entire duration of Ca(NO_3_)_2_ stress.

**FIGURE 2:**
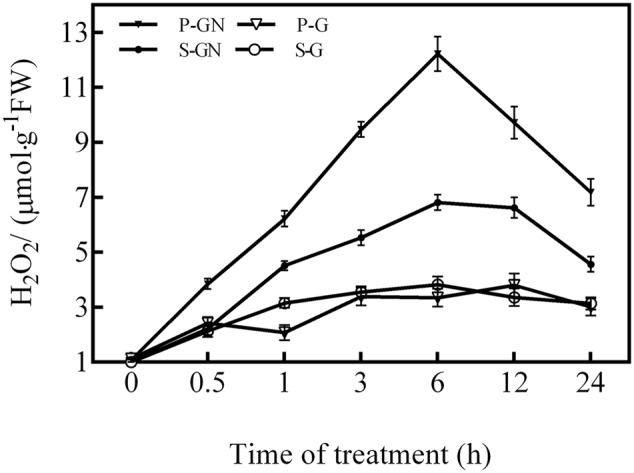
**Time course of changes in the H_2_O_2_ content in the leaves of self-grafted and pumpkin-grafted cucumber seedlings exposed to 80 mM Ca(NO_3_)_2_.** Each value is the mean ± SE of six independent experiments (*n* = 6). P-G, pumpkin-grafted cucumber seedlings grown in Hoagland’s solution; P-GN, pumpkin-grafted cucumber seedlings with 80 mM Ca(NO_3_)_2_; S-G, self-grafted cucumber seedlings grown in Hoagland’s solution; S-GN, self-grafted cucumber seedlings with 80 mM Ca(NO_3_)_2_.

The H_2_O_2_ content in the leaves of pumpkin-grafted and self-grafted cucumber seedlings increased by Ca(NO_3_)_2_ stress compared to the corresponding controls (**Figure 2**). The H_2_O_2_ content increased after 0.5 h of Ca(NO_3_)_2_ stress and peaked at 6 h, after which it rapidly decreased in the leaves of pumpkin-grafted and self-grafted cucumber seedlings. However, the pumpkin-grafted seedlings had a higher H_2_O_2_ level than that of the self-grafted seedlings leaves during the treatment of Ca(NO_3_)_2_ stress. Under non-saline conditions, there were no significant differences in the H_2_O_2_ level between self-grafted seedlings and pumpkin-grafted cucumber leaves.

### Effects of Ca(NO_3_)_2_ Stress on the Activities of Antioxidant Enzymes in the Leaves of Pumpkin-Grafted and Self-Grafted Cucumber Seedlings

**FIGURE 3:**
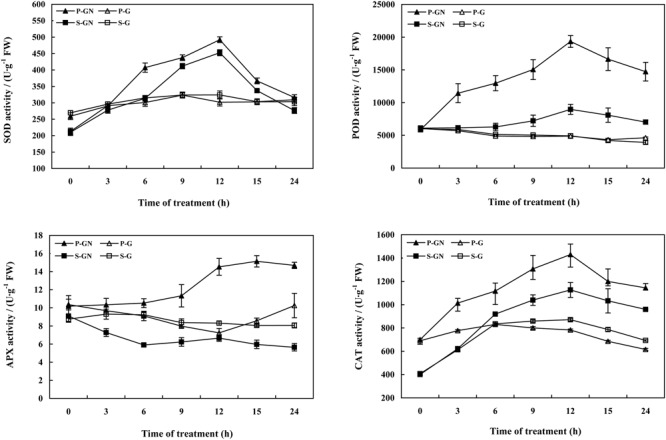
**Time course of changes in the activities of SOD, POD, CAT, and APX in the leaves of self-grafted and pumpkin-grafted cucumber seedlings exposed to 80 mM Ca(NO_3_)_2_.** Each value is the mean ± SE of six independent experiments (*n* = 6). P-G, pumpkin-grafted cucumber seedlings grown in Hoagland’s solution; P-GN, pumpkin-grafted cucumber seedlings with 80 mM Ca(NO_3_)_2_; S-G, self-grafted cucumber seedlings grown in Hoagland’s solution; S-GN, self-grafted cucumber seedlings with 80 mM Ca(NO_3_)_2_.

Treatment with Ca(NO_3_)_2_ stress led to similar changes of in the antioxidant enzyme activities in the leaves of pumpkin-grafted and self-grafted cucumber seedlings, but the changes in the activities of antioxidant enzymes in the rootstock-grafted leaves were more significant and the range was much wider than those of the self-grafted seedlings (**Figure 3**). Compared to their corresponding controls, the activities of SOD, POD and CAT increased in the leaves of pumpkin-grafted and self-grafted cucumber seedlings under Ca(NO_3_)_2_ stress. Moreover, these values of the pumpkin-grafted seedlings were increasingly higher than those of the self-grafted seedlings under Ca(NO_3_)_2_ stress. The activities of SOD, POD and APX in the pumpkin-grafted seedlings leaves increased after 6 h of Ca(NO_3_)_2_ stress and peaked at 12 h, after which it gradually decreased. The activity of CAT in the leaves increased and peaked at 12 h, then decreased at 24 h, and then increased until 48 h in pumpkin-grafted and self-grafted cucumber seedlings under Ca(NO_3_)_2_ stress (Gao et al., 2015). Under non-saline conditions, there was no significant difference in the activities of antioxidant enzymes between pumpkin-grafted and self-grafted seedlings leaves, except for the APX activity at 12 and 36 h.

### ABA-Mediated Accumulation of H_2_O_2_ in the Leaves of Pumpkin-Grafted Seedlings under Ca(NO_3_)_2_ Stress

**FIGURE 4:**
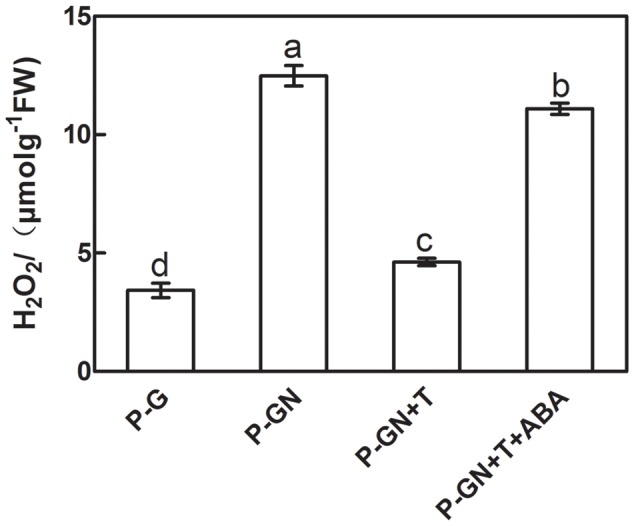
**Effects of pretreatment with the ABA inhibitor sodium tungstate (T) and exogenous ABA on the content of H_2_O_2_ in the leaves of pumpkin-grafted cucumber seedlings exposed to 80 mM Ca(NO_3_)_2_ stress.** The pumpkin-grafted cucumber seedlings were pretreated with 5 mM T (P-GN + T) and 100 μM exogenous ABA (P-GN + T + ABA) for 8 and 12 h, respectively, and then exposed to 80 mM Ca(NO_3_)_2_ for 12 h. Each histogram represents a mean value of three independent experiments, and the vertical bars indicate SE (*n* = 6). Different letters indicate significant differences at *P < 0.05*, according to Duncan’s multiple range tests. P-G, pumpkin-grafted cucumber seedlings grown in Hoagland’s solution; P-GN, pumpkin-grafted cucumber seedlings with 80 mM Ca(NO_3_)_2_.

Sodium tungstate (T), an ABA synthesis inhibitor, and exogenous ABA were used to determine whether ABA is involved in inducing H_2_O_2_ accumulation in pumpkin-grafted cucumber seedlings under Ca(NO_3_)_2_ stress. Ca(NO_3_)_2_ stress significantly increased the H_2_O_2_ content of the pumpkin-grafted seedlings leaves compared to that of the control, but pretreatment with 5 mM sodium tungstate remarkably inhibited the increased H_2_O_2_ induced by Ca(NO_3_)_2_ stress. However, the application of exogenous 100 μM ABA to Ca(NO_3_)_2_ stress alleviated the inhibition of the H_2_O_2_ content in the leaves of the pumpkin-grafted seedlings with sodium tungstate (**Figure 4**).

### ABA Involved in Enhancing Antioxidant Capacity in Pumpkin-Grafted Seedlings Leaves under Ca(NO_3_)_2_ Stress

**FIGURE 5:**
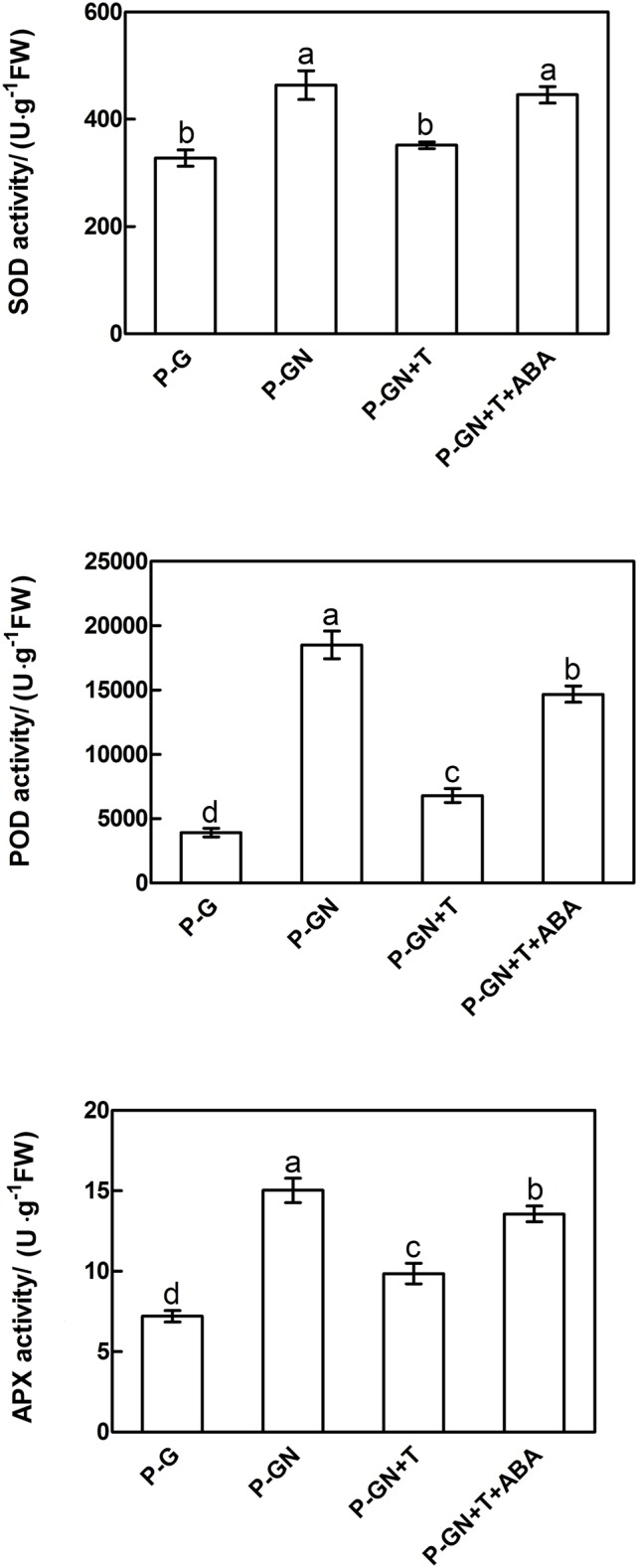
**Effects of pretreatment with the ABA inhibitor sodium tungstate (T) and exogenous ABA on the activities of SOD, POD and APX in the leaves of pumpkin-grafted cucumber seedlings exposed to mM Ca(NO_3_)_2_.** The pumpkin-grafted cucumber seedlings were pretreated with 5 mM T (P-GN + T) and 100 μM exogenous ABA (P-GN + T + ABA) for 8 and 12 h, respectively, and then exposed to 80 mM Ca(NO_3_)_2_ for 12 h. Each histogram represents the mean value of three independent experiments, and the vertical bars indicate SE (*n* = 6). Different letters indicate significant differences at *P < 0.05*, according to Duncan’s multiple range tests. P-G, pumpkin-grafted cucumber seedlings grown in Hoagland’s solution; P-GN, pumpkin-grafted cucumber seedlings with 80 mM Ca(NO_3_)_2_.

**FIGURE 6:**
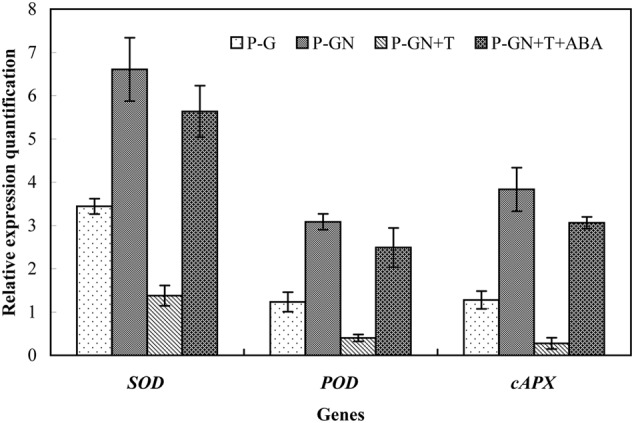
**Effects of pretreatment with the ABA inhibitor sodium tungstate (T) and exogenous ABA on the expression of *SOD, POD*, and *APX* in the leaves of pumpkin-grafted cucumber seedlings exposed to 80 mM Ca(NO_3_)_2_.** The pumpkin-grafted cucumber seedlings were pretreated with 5 mM T (P-GN + T) and 100 μM exogenous ABA (P-GN + T + ABA) for 8 and 12 h, respectively, and then exposed to 80 mM Ca(NO_3_)_2_ for 12 h. Each histogram represents the mean value of three independent experiments, and the vertical bars indicate SE (*n* = 3). P-G, pumpkin-grafted cucumber seedlings grown in Hoagland’s solution; P-GN, pumpkin-grafted cucumber seedlings with 80 mM Ca(NO_3_)_2_.

Abscisic acid synthesis inhibitor T was used to determine the effects of ABA on the antioxidant defense of pumpkin-grafted cucumber seedlings under Ca(NO_3_)_2_ stress. According to the results in **Figure 3**, an irregular change in CAT activity was induced by Ca(NO_3_)_2_ stress; thus, we only studied the other three enzymes. Pretreatment with sodium tungstate significantly inhibited the increase in the SOD, POD and APX activities in the leaves of the pumpkin-grafted seedlings under Ca(NO_3_)_2_ stress (**Figure 5**). However, exogenous ABA in addition to Ca(NO_3_)_2_ stress alleviated the inhibition of the SOD, POD and APX activities of the pumpkin-grafted seedlings in the presence of sodium tungstate. We analyzed the expression profiles of three transcripts that encoded SOD, POD and APX using real-time quantitative RT-PCR to evaluate the correlation between these enzyme activities and their encoded gene expression after 12 h of Ca(NO_3_)_2_ stress (**Figure 6**). The *SOD, POD*, and *cAPX* expression patterns were similar to their enzyme activities under different treatment conditions.

### H_2_O_2_ Accumulation Induced Antioxidant Defense in Pumpkin-Grafted Seedlings Leaves under Ca(NO_3_)_2_ Stress

**FIGURE 7:**
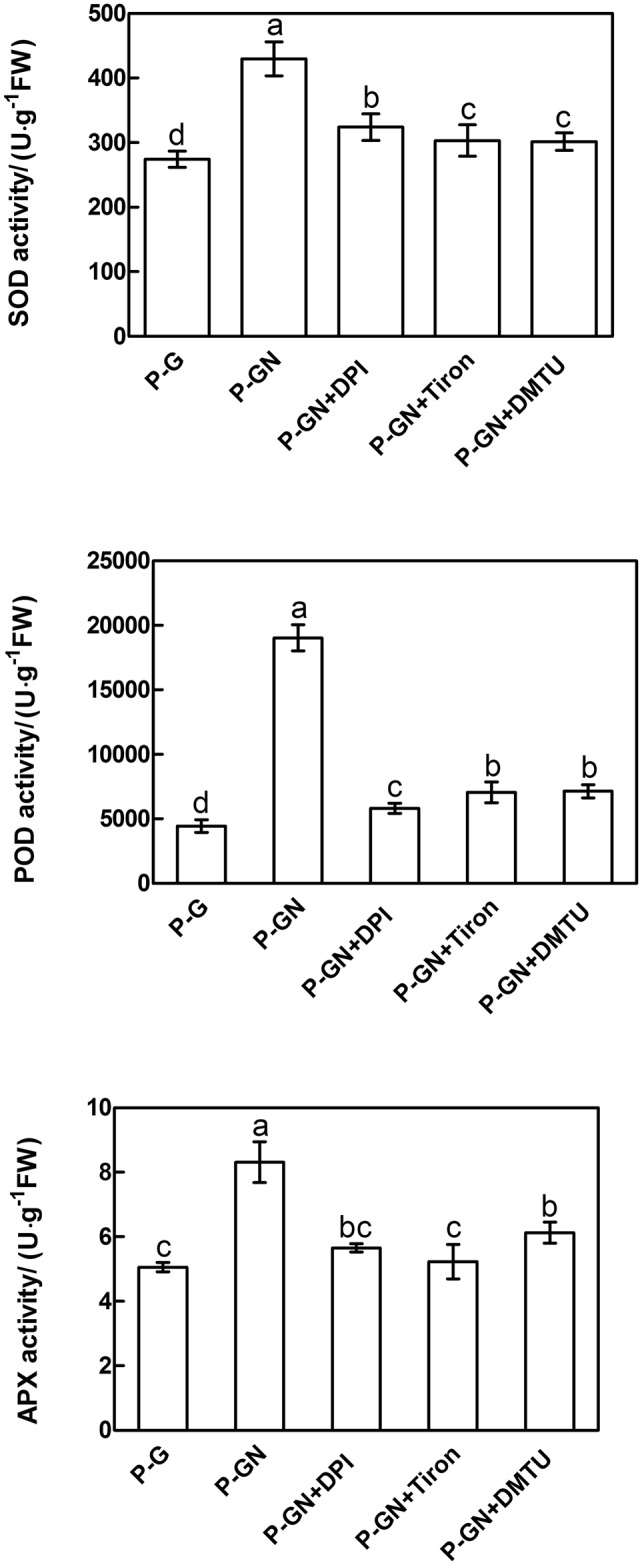
**Effects of pretreatment with the NADPH oxidase inhibitor diphenyleneiodonium (DPI), the $O_{2}^{–}$ scavenger Tiron and the H_2_O_2_ scavenger DMTU on the activities of SOD, POD, and APX in the leaves of pumpkin-grafted cucumber seedlings exposed to 80 mM Ca(NO_3_)_2_.** The pumpkin-grafted cucumber seedlings were pretreated with 100 μM DPI (P-GN + DPI), 10 mM Tiron (P-GN + Tiron), and 5 mM DMTU (P-GN + DMTU) for 8 h. Each histogram represents the mean value of three independent experiments, and the vertical bars indicate SE (*n* = 6). Different letters indicate significant differences at *P < 0.05*, according to Duncan’s multiple range tests. P-G, pumpkin-grafted cucumber seedlings grown in Hoagland’s solution; P-GN, pumpkin-grafted cucumber seedlings with 80 mM Ca(NO_3_)_2_.

**FIGURE 8:**
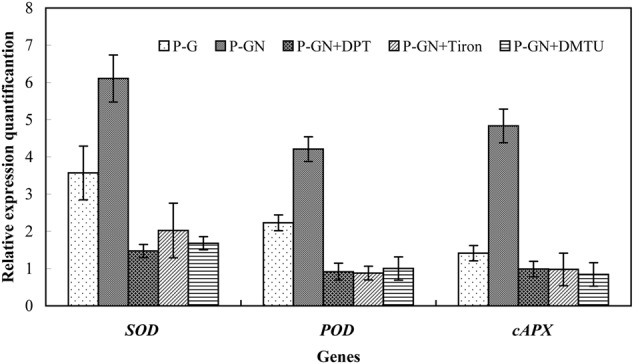
**Effects of pretreatment with the NADPH oxidase inhibitor diphenyleneiodonium (DPI), the $O_{2}^{–}$ scavenger Tiron and the H_2_O_2_ scavenger DMTU on the expression of the *SOD, POD*, and *cAPX* in pumpkin-grafted cucumber seedlings leaves exposed to 80 mM Ca(NO_3_)_2_.** The pumpkin-grafted cucumber seedlings were pretreated with 100 μM DPI (P-GN + DPI), 10 mM Tiron (P-GN + Tiron), and 5 mM DMTU (P-GN + DMTU) for 8 h. Each histogram represents the mean value of three independent experiments, and the vertical bars indicate SE (*n* = 3). P-G, pumpkin-grafted cucumber seedlings grown in Hoagland’s solution; P-GN, pumpkin-grafted cucumber seedlings with 80 mM Ca(NO_3_)_2_.

Reactive oxygen species are key signaling molecules for stress tolerance in plants. We thus examined the effects of pretreatment with ROS manipulators (DPI, Tiron and DMTU) on the gene expression and activities of antioxidant enzymes in pumpkin-grafted seedling leaves. Ca(NO_3_)_2_ stress induced a significant increase in the activities of SOD, POD and APX and their corresponding gene expression in pumpkin-grafted cucumber leaves, and these positive effects were completely prevented by pretreatment with DPI, Tiron and DMTU (**Figures 7** and **8**). These results indicate that ABA is involved in H_2_O_2_-accumulation-mediated antioxidant defense in pumpkin-grafted plants and in the subsequently improved Ca(NO_3_)_2_ tolerance of cucumber seedlings.

## Discussion

It is well known that grafting with stress-tolerant rootstock can enhance plant tolerance to stress. In our previous studies, the Ca(NO_3_)_2_ stress tolerance of cucumber seedlings could be enhanced by grafting with pumpkin from different physiological aspects, including osmotic adjustment ability, nitrogen metabolism, soluble protein expression and antioxidant defense (Wang et al., 2012; Xing et al., 2015), but little information is available about the roles of the ABA and H_2_O_2_ signaling pathways in relieving Ca(NO_3_)_2_ stress. In this study, we present evidence that grafting-induced ABA accumulation in cucumber leaves triggered H_2_O_2_ production, thus enhancing activities of antioxidant enzymes, the expression of their encoding gene and the subsequent salt tolerance in response to Ca(NO_3_)_2_ stress.

Abscisic acid is the most important phytohormone that has multiple functions in the developmental processes of plants and enhances plants tolerance to various stresses including salinity, drought, and low temperature (Giraudat et al., 1994; Sah et al., 2016). In this study, the ABA content greatly increased in both self-grafted and rootstock-grafted cucumber leaves during the Ca(NO_3_)_2_ treatment (**Figure 1**). The increased ABA levels by grafting under stressed conditions probably resulted not only from the increased catabolism via the mevalonic acid-independent pathway but also from the translocation of other tissues or organs (Roychoudhury et al., 2013). Moreover, we also observed that the increased ABA content in the leaves of the rootstock-grafted plants was higher than that of the self-grafted plants under Ca(NO_3_)_2_ stress. These results may indicate that grafting cucumber seedlings with pumpkin rootstock exhibited a strong ability to resist Ca(NO_3_)_2_ stress. It has also been shown that increased endogenous ABA activates a complex signaling network leading to cellular responses to stresses (Ng et al., 2014). Several studies have shown that ABA accumulation induced by abiotic stresses could have a physiological effect on ROS production (Pei et al., 2000; Zhang et al., 2006).

As secondary messengers, ROS mediate variety of physiological functions and defense responses against abiotic stresses in plants. These functions include the regulation of seed germination (Roach et al., 2010), root development (Foreman et al., 2003), photosynthesis (Foyer et al., 2012), senescence (Gao et al., 2016), and adaptive responses to abiotic stresses (Jiang and Zhang, 2002a,b; Saxena et al., 2016). H_2_O_2_, a type of ROS, generates rapidly in plants in response to stress conditions (Saxena et al., 2016). However, it is not clear whether there exist similar responses in grafted plants, especially in plants exposed to Ca(NO_3_)_2_ stress. In this study, our results showed that Ca(NO_3_)_2_ stress increased the ABA and H_2_O_2_ contents and the antioxidant defense of pumpkin-grafted cucumber leaves. It is essential for plants to maintain the interaction between ABA and H_2_O_2_ to avoid any oxidative stress induced by adverse environmental factors (Saxena et al., 2016). As the time course of the production of ABA (**Figure 1**) and H_2_O_2_ (**Figure 2**) showed, the peak time of ABA production (3 h) preceded that of H_2_O_2_ production (6 h); then, at 12 h of Ca(NO_3_)_2_ treatment, the activities of the antioxidant enzymes SOD, POD and APX peaked. It was hypothesized that the antioxidant defense induced by Ca(NO_3_)_2_ stress in the leaves of pumpkin-grafted cucumber plants was initiated by ABA and involved H_2_O_2_.

To test our hypothesis, subsequent experiments with different inhibitors and scavengers, such as the ABA inhibitor sodium tungstate (T) and the H_2_O_2_ scavenger DMTU, were performed. Our results showed that the increased H_2_O_2_ content under Ca(NO_3_)_2_ stress was blocked in the pumpkin-grafted cucumber leaves that were pretreated with T (**Figure 4**). However, the decrease in the H_2_O_2_ level of the pumpkin-grafted plants with T recovered by the application of exogenous ABA. These results suggest a crosslink between ABA and H_2_O_2_ signaling pathways. Previous studies have also clearly demonstrated that the increased H_2_O_2_ levels depend on the activation of ABA in pumpkin-grafted cucumber leaves under Ca(NO_3_)_2_ stress. Similar result was observed by Guan et al. (2000), who showed that endogenous H_2_O_2_ level significantly increased under high concentrations of ABA conditions. In addition, the antioxidant defense of leaves induced by Ca(NO_3_)_2_ treatment was significantly inhibited in pumpkin-grafted cucumber plants that were pretreated with T (**Figure 5**). T blocks the formation of ABA from abscisic aldehyde by impairing abscisic aldehyde oxidase (Hansen and Grossmann, 2000). These results suggest that ABA was required for the increased Ca(NO_3_)_2_ stress-induced H_2_O_2_ production and antioxidant defense in grafted cucumber plants.

It has been shown that ABA-induced ROS production increases the activities of SOD, CAT, APX and GR in maize (Jiang and Zhang, 2002a,b). Recent studies have determined that ABA and H_2_O_2_ induced activation of antioxidant enzymes by using transgenic tobacco plants in combination with their inhibitors or scavengers (Lu et al., 2014). In the present study, pretreatment with several ROS manipulators, such as the NADPH oxidase inhibitor DPI, the $O_{2}^{•–}$ scavenger Tiron and the H_2_O_2_ scavenger DMTU, almost completely depressed ABA-induced antioxidant defense in the leaves of pumpkin-grafted cucumber plants under Ca(NO_3_)_2_ stress (**Figure 6**). In the leaves of maize seedlings, NADPH oxidase is involved in ABA-induced ROS production (Jiang and Zhang, 2002b). This may induce oxidative damage to plant cells, resulting in disrupted metabolic function and destroyed cellular integrity (Ozden et al., 2009). H_2_O_2_ generation induced by NADPH oxidase might be as a reaction cascade that triggers the antioxidant enzyme activities in *Arabidopsis thaliana*, thereby mitigating the salt stress-induced oxidative damage (Rejeb et al., 2015). According to the review by Forman (2007), H_2_O_2_ can be toxic to plants but can also be an important stress signal. H_2_O_2_ can be synthesized in response to exogenous ABA. H_2_O_2_ mediates, at least in part, ABA responses, including defense mechanisms, stomatal closure and gene expression (Guan et al., 2000; Pei et al., 2000; Saxena et al., 2016). Desikan et al. (2001) provided further evidence of H_2_O_2_ as a central signaling mediator of the abiotic stress response in plants by using cDNA microarray technology. Their studies showed that oxidative stress induced the expression of some genes, such as *SLN1-SSK1* (a gene encoding a potential hybrid His kinase) and that *MAPKKs* (MAPK kinases) are up-regulated by H_2_O_2_. This evidence suggests that H_2_O_2_ was involved in the ABA-induced antioxidant defense in the leaves of pumpkin-grafted seedlings, thus enhancing cucumber tolerance in responses to Ca(NO_3_)_2_ stress.

## Conclusion

**FIGURE 9:**
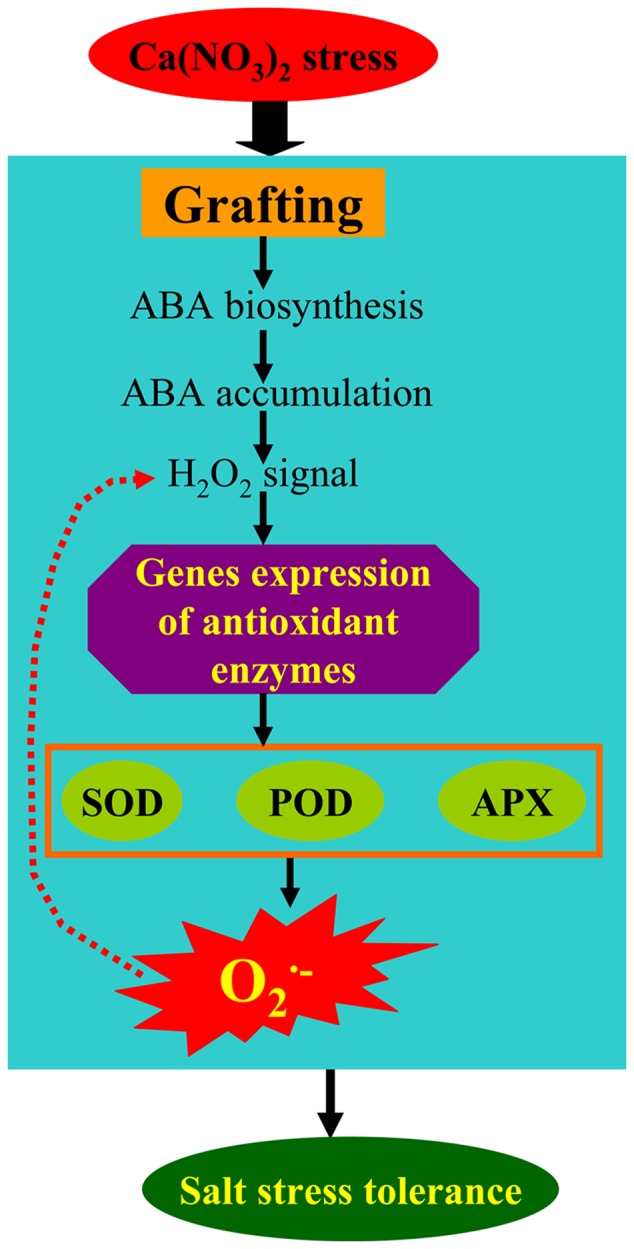
**A simplified metabolic scheme and signal transduction pathway for ABA and H_2_O_2_ in the leaves of pumpkin-grafted cucumber seedlings exposed to 80 mM Ca(NO_3_)_2_.** The model is based on the results presented here. The red arrow (dotted line) indicates possible metabolic pathways in our experiment.

In the present study, our results indicate that the ABA responses of pumpkin-grafted and self-grafted cucumber leaves were differently induced by 80 mM Ca(NO_3_)_2_ stress. The accumulation of ABA was involved in the rapid accumulation of H_2_O_2_, and the accumulation of H_2_O_2_ induced the activities of SOD, POD and APX and the expression of their encoded genes in pumpkin-grafted cucumber leaves (**Figure 9**). The higher capacity of antioxidant defense in the pumpkin-grafted cucumber plants induced by the ABA signaling pathways presented in our studies may be part of the reason for the better performance of these plants than that of self-grafted cucumber plants under Ca(NO_3_)_2_ stress (**Supplementary Figure S1**). The specific mechanism of ABA-H_2_O_2_ signaling requires further investigation to obtain more insight into the root-shoot signaling in rootstock-grafted cucumber plants.

## Author Contributions

SS wrote the main manuscript text. PG, LL and YY prepared all figures and modified this manuscript until submitted. JS performed the experiments. SG designed the research and proposed the research proceeding. All authors reviewed the manuscript.

## Conflict of Interest Statement

The authors declare that the research was conducted in the absence of any commercial or financial relationships that could be construed as a potential conflict of interest.

## Footnotes

1 http://cucumber.genomics.org.cn

## Supplementary Material

FIGURE S1 **Phenotypic response of self-grafted and pumpkin-grafted cucumber seedlings exposed to 80 mM Ca(NO3)2 for 7 days.** P-G, pumpkin-grafted cucumber seedlings grown in Hoagland’s solution; P-GN, pumpkin-grafted cucumber seedlings with 80 mM Ca(NO_3_)_2_; S-G, self-grafted cucumber seedlings grown in Hoagland’s solution; S-GN, self-grafted cucumber seedlings with 80 mM Ca(NO_3_)_2_.

## Abbreviations

ABA: abscisic acid
APX: ascorbate peroxidase
CAT: catalase
DMTU: dimethylthiourea
DPI: diphenyleneiodonium chloride
POD: peroxidase
SOD: superoxide dismutase
Tiron: 1,2-dihydroxybenzene- 3,5-disulphonic acid
