# Supplementary figures and images for: Abscisic Acid-Induced H2O2 Accumulation Enhances Antioxidant Capacity in Pumpkin-Grafted Cucumber Leaves under Ca(NO3)2 Stress
Source: Front Plant Sci. 2016 Sep 30;7:1489. doi: 10.3389/fpls.2016.01489 (PMC5043297; doi:10.3389/fpls.2016.01489)

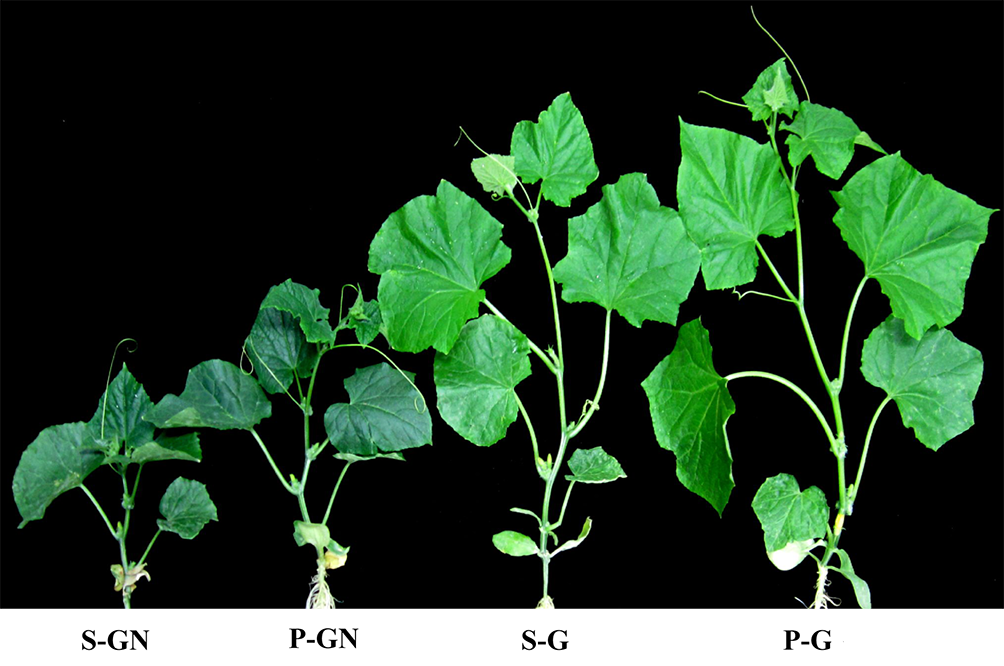

Supplement: FIGURE S1 — Phenotypic response of self-grafted and pumpkin-grafted cucumber seedlings exposed to 80 mM Ca(NO3)2 for 7 days. P-G, pumpkin-grafted cucumber seedlings grown in Hoagland’s solution; P-GN, pumpkin-grafted cucumber seedlings with 80 mM Ca(NO3)2; S-G, self-grafted cucumber seedlings grown in Hoagland’s solution; S-GN, self-grafted cucumber seedlings with 80 mM Ca(NO3)2. [file Image_1.TIF]
